# Exploring the educational journey: perspectives of ethnic minority GP-trainees in Dutch GP-specialty training - a qualitative interview study

**DOI:** 10.1186/s12939-024-02341-x

**Published:** 2024-11-28

**Authors:** N.M van Moppes, M. Nasori, A.C. Jorissen, J.M. van Es, J. Bont, M.R.M. Visser, M.E.T.C. van den Muijsenbergh

**Affiliations:** 1 Department of General Practice and Public Health Research Institute, Amsterdam UMC location AMC, Meibergdreef 9,, Amsterdam, AZ 1105 the Netherlands; 2 Department of General Practice, Radboud University Medical Center, Nijmegen, the Netherlands

**Keywords:** GP-specialty training, Diversity, Inclusion, Equity, Postgraduate medical education, Inclusive learning environment

## Abstract

**Background:**

Previous research highlights persistent differential attainment by ethnicity in medical education, wherein the perceived inclusiveness significantly influences ethnic minority students’ and trainees’ outcomes. Biased organizational practices and microaggressions exacerbate the challenges faced by ethnic minorities, leading to lower academic performance and higher dropout rates. Consequently, understanding ethnic minority GP-trainees’ experiences and perspectives regarding relevant educational aspects is crucial for addressing these disparities and cultivating a more inclusive environment within medical education.

**Research question:**

We aimed to investigate the experiences of minority GP-trainees throughout their educational journey in Dutch GP-specialty training, emphasizing their challenges, sources of support, and suggestions for enhancing their learning environment.

**Method:**

We conducted semi-structured, in-depth interviews with minority GP trainees, employing purposive convenience sampling to ensure diversity across multiple dimensions. These included gender, age, ethnicity, social background, migration generation, educational stage, encountered challenges, sources of support, and the GP training institute attended. The analysis involved iterative, open and axial coding, followed by generating, reviewing, and defining themes. For a structured analysis of encountered microaggressions, we adopted Sue's Taxonomy of Microaggressions.

**Results:**

All fourteen ethnic minority interviewees had faced educational barriers stemming from misunderstandings and stereotyping in a predominantly 'white' organization. These barriers impacted various aspects of their education, including professional identity formation, application, admission, assessment procedures, social networks, course content, and expert guidance. Microaggressions permeated throughout their educational journey, hindering their full expression and potential. Their ideal GP-specialty training emphasized uniqueness of all trainees, comprehensive staff engagement in inclusivity, robust diversity, equity, and inclusion (DEI)-policies, individual mentorship, transparent standards, concise language usage in test questions, and bias elimination through mandatory DEI staff training.

**Conclusion:**

Ethnic minority GP-trainees in the Netherlands face significant challenges like biased assessment and admission, stereotyped course content, inadequate support networks, and microaggressions, putting them at risk for underperformance outcomes. They emphasize the need for inclusive training with robust DEI-policies to eliminate bias.

**Supplementary Information:**

The online version contains supplementary material available at 10.1186/s12939-024-02341-x.

## Introduction

 Global migration has diversified healthcare demographics [1–4], emphasizing the importance of ethnically diverse General Practitioners (GPs) for improved healthcare outcomes, especially when it concerns underserved migrant populations [5, 6]. This emphasis is particularly critical in the Netherlands, where GPs serve as healthcare gatekeepers. Therefore, understanding the perspectives of ethnic minority trainees in Dutch GP-specialty training is crucial for sustainably addressing diverse healthcare needs.

However, international studies reveal persistent *differential attainment by ethnicity* in medical education, with minority groups achieving lower outcomes compared to their majority counterparts [7–10]. This attainment gap extends beyond socio-demographic and learner-related factors [10, 11], and it persists after controlling for pre-university academic performance, socioeconomic status, language proficiency, motivation, study habits, living situation, and personality [12, 13].

Increased awareness of these ethnic inequalities prompted medical schools to reassess challenges faced by minority students and trainees [14–16], shifting from the *student deficit model* to *institutional change research* [17]. Previous studies highlight social factors, including interaction quality and perceived inclusiveness, affecting minority students’ academic outcomes [17–20]. Implicit social boundaries, embedded in normalized organizational rules, and unintentional bias favoring white majority cultures identify as barriers for ethnic minority individuals across career stages, elevating their risk of being assessed as underperforming [11, 16, 18–20].

Institutional ethnic discrimination stemming from biased policies and practices [19] harms students’ well-being [21–23], increasing their risk of academic underperformance and dropout [24]. Subtle manifestations, including microaggressions and stereotyping, seem to have a more pronounced impact on academic outcomes, attrition rates, and professional opportunities than overt discrimination [15, 25, 26]. Also, trainees’ sociocultural backgrounds, even in later migrant generations, can affect communication styles [27], potentially leading to biased assessments by assessors unfamiliar with non-dominant styles [28].

In addition to these identity and professionalism dynamics, medical education institutions encompass unique vulnerabilities regarding diversity, equity, and inclusion (DEI) across application and admission [29–32], support networks [33, 34], course content, expert guidance [35, 36], and assessments [37]. Microaggressions permeate these structures, hindering minority trainees from fully expressing themselves professionally [15, 18, 23, 33, 38–40] and squandering valuable learning time [41–43].

Fostering inclusive learning environments across all these educational aspects is essential for supporting students and trainees to their full potential. However, learning environments are complex and shaped by multiple stakeholders’ perceptions in formal and informal contexts [44–46]. Inclusive interventions drive cultural change within educational institutes, demanding the development of new knowledge and professional identities [16] amid challenges of discomfort and questioning deemed neutral practices and norms [47]. Theories on inclusiveness highlight institutional policies, personal perceptions of being valued for unique perspectives, and a sense of belonging [48].

While numerous studies describe minority undergraduate medical students’ perspectives on learning environments [18, 20, 49, 50], limited research addresses those of minority postgraduate medical trainees [36, 51]. Recent findings indicate that minority undergraduate medical students experience less supportive learning environments, weaker social networks, and lower social capital than their majority peers [16, 18, 52]. They face challenges related to cultural understanding, identity, racism, and microaggressions, leading to burnout, depressive symptoms, isolation, self-doubt, and restricted professional identity formation and learning opportunities [15, 23]. Similar outcomes were observed among clinical and psychiatry residents [36, 51].

While valuable, existing studies on educational barriers for medical students and clinical residents may overlook the dynamics of postgraduate medical learning in GP-specialty training. Unlike undergraduate lecture halls and postgraduate clinical settings with multiple assessors, GP-trainees primarily learn through one-on-one supervised in-clinic training supplemented by small-group, in-faculty training once a week. This blend emphasizes experiential learning and demands specific skills from trainees, in-faculty teachers, and in-clinic supervisors [46]. Consequently, postgraduate GP-trainees’ experiences may differ from those of their undergraduate and clinical peers.

Understanding ethnic minority GP trainees’ perspectives on their educational journey is crucial for addressing the attainment gap and promoting inclusiveness in GP-specialty training [52–55]. Therefore, we explored their ideas and experiences throughout Dutch GP-specialty training, and their views on ideal GP-specialty training.

### Study objectives

Our objective was to investigate the experiences of minority GP-trainees throughout their educational journey in the Dutch GP-specialty training program, emphasizing their challenges, sources of support, and suggestions for enhancing the overall learning environment.

## Methods

### Design

In this qualitative study, we conducted semi-structured, in-depth interviews with minority GP-trainees.

### Setting

We conducted this study within the eight GP-specialty training institutes of the GP-specialty training Netherlands network (HN). In the Netherlands, one in three medical graduates aspires to enter GP-specialty training, with 921 accepted new trainees in 2023 and an anticipated acceptance of 1,190 in 2024 distributed across the eight training institutes. While approximately 28% of the Dutch population under age 25 belongs to ethnic minority groups, they seem underrepresented among GP-trainees, comprising around 15%, of whom many completed pre-training at Dutch Medical Schools [9]. Nationally centralized entry assessments evaluate applicants’ medical knowledge, motivation, and Dutch proficiency. Dutch GP-specialty training is a three-year dual-track program combining hands-on clinical experience with one-day-a-week in-faculty education. It aims for a supportive learning environment offering a trusted social network and expert guidance with well-balanced DEI-related course content. The program incorporates protocolled assessments, including annual knowledge tests, consultation observations with reflective reports, systematic competence assessment lists, and reviews of learning objectives.

### Participants

We included GP-trainees who self-identified as belonging to ethnic minority groups and were currently enrolled or graduated within the last five years from one of the eight Dutch GP-specialty training institutes. Utilizing purposive convenience sampling and snowballing techniques, we strived for a comprehensive range of perspectives, seeking maximum diversity regarding gender, age, ethnic and social backgrounds, generation of migration, educational stage, experienced challenges and sources of support, and the specific GP-training institute attended. We emailed eligible participants additional information and scheduled interview appointments upon receiving informed consent for anonymized demographic and interview data collection and publication, with no financial compensation provided for participation.

### Data collection and analysis

This study was part of a broader project exploring minority GP-trainees’ experiences, perspectives, and coping strategies. Data collection and analysis occurred iteratively from April 2019 to October 2023, involving in-depth interviews of 49–86 min by five trained interviewers (MN, NMvM, AJ, SZ, and SG) with the latter two supervised by the three primary interviewers (MN, NMvM, and AJ).

Before the interview, interviewers introduced the study’s context, ensuring confidentiality. They then initiated the exchange of ideas with broad, open questions, allowing ample space for participants’ experiences and emotions, in line with a reflexive thematic analytical approach [56, 57]. To delve deeper into participants’ experiences, we utilized a topic list (Appendix) informed by existing literature and expert input while accommodating spontaneous questions and additions. Additionally, we employed identity cards [58], inspired by Ngozi Ardichie’s 2009 TED talk on multifaceted identities [59]: nineteen identity cards with eighteen representing unique identity aspects (e.g., gender, life stage, family position, migration history), and one blank for personal interpretation. The interviewer invited participants to select three cards that most influenced their professional development.

Following each interview, the interviewer compiled a reflection report, assessing the atmosphere and conduct. Audio recordings were transcribed verbatim and anonymized.

Using MAXQDA software, version 12, four researchers (MN, MV, NM, and AJ) initiated open coding by associating interviewees’ expressions and comments with keywords. They discussed their encodings until consensus. Then, MN, MV and AJ autonomously proceeded with axial and thematic analysis, using the coded keywords as guidance, involving a fourth researcher (NM) in unresolved unclarities or disagreements [60]. This coding process iteratively shifted between open, axial, and selective coding until we identified structural key categories with no new topics or categories emerging, indicating data saturation. Initially refraining from a specific analytical framework, we familiarized ourselves with the material, identifying recurring themes and patterns in participants’ perspectives and perceptions of the educational environment [56, 57].

**Fig. 1:**
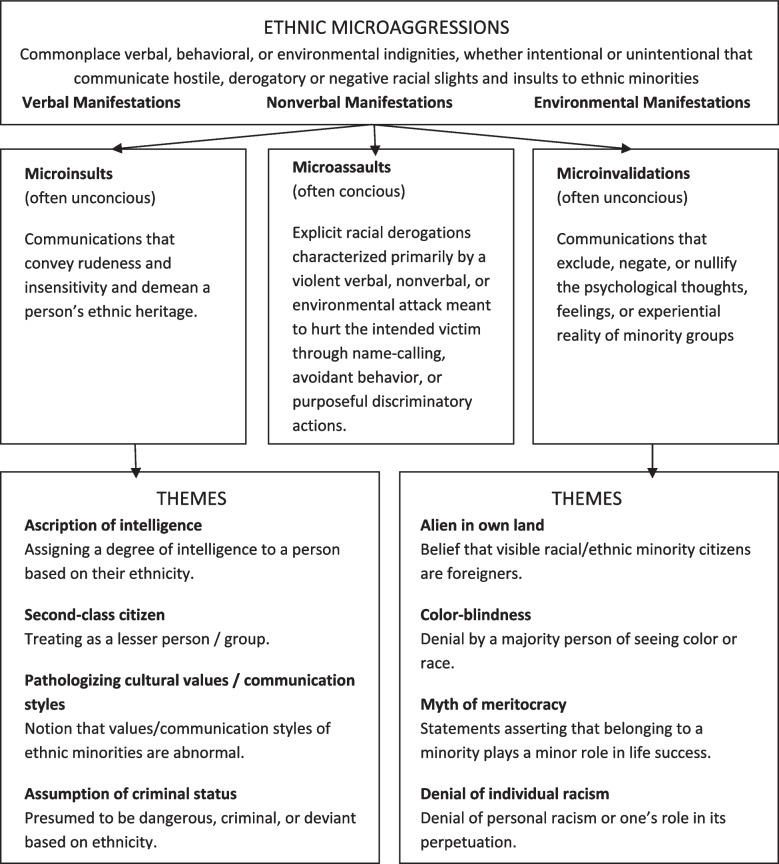
Categories and interrelationships of microagressions

Building on existing literature, our analysis focused on participants’ professional identities, their unique experiences within various educational settings [30, 32, 34–37], and encountered microaggressions (defined as day-by-day subtle, often unintended slights directed at marginalized groups, manifesting both verbally and nonverbally) [15, 18, 23, 39–42, 61]. For a structured analysis, we adopted Sue’s Taxonomy of Microaggressions, which delineates three distinct types: microassaults, microinsults, and microinvalidations [62]. These categories encapsulate overt, covert, and hidden offensive messages with varying levels of perpetrator awareness (Fig. 1) [61].

### Reflexivity and ethics

This research follows our preceding quantitative study indicating an increased risk of underperformance assessments among minority GP trainees compared to their majority peers [9]. The research team consisted of three GPs (PhD candidate (NM), head of the GP department (JB), and professor in health disparities (MM)), a cognitive psychologist (MV), a research assistant MN), and a GP-trainee (AJ), affiliated with Dutch GP research and training institutes, all experienced in medical education research. None of the researchers or interviewers had direct teaching or assessment roles with the participants.

Two researchers (NM and MN) identify as minority females. While their backgrounds may heighten sensitivity to how minority peers are addressed, a risk exists of experiences resonating with them more prominently. To mitigate this potential, we held reflective sessions, addressing diverse viewpoints and emphasizing the researchers’ roles as instruments in data collection and analysis. These sessions involved questioning each other on experiences, expectations, preoccupations, and opinions that may have influenced the process.

## Results

Fourteen GP-trainees across eight Dutch GP-specialty training institutes participated. They included nine females and five males, mostly aged between 27 and 33, with one outlier at 40. Among them, eight were in various stages of training (one in year 1, two in year 2, and five in year 3), while five had recently completed and one had discontinued their GP specialty training (Table 1). The participants represented diverse educational outcomes, ranging from successful graduates and honors program participants to those in mandatory coaching or involuntary dropout situations. Eight were second-generation, and six were first-generation migrants who had moved to the Netherlands 8–26 years ago.

**Table 1:** Participants

Participant	Gender	Trainee / Alum	Migration background
1	M	Trainee	First-generation
2	F	Trainee	First-generation
3	F	Trainee	Second-generation
4	F	Alum	Second-generation
5	M	Alum	First-generation
6	F	Trainee	First-generation
7	F	Trainee	Second-generation
8	M	Trainee	Second-generation
9	F	Alum	First-generation
10	F	Alum	First-generation
11	M	Dropped out	Second-generation
12	F	Trainee	Second-generation
13	F	Alum	Second-generation
14	M	Trainee	Second-generation

Our participants’ responses revealed seven key themes, reflecting the complex dynamics shaping their educational journeys and professional aspirations in GP-specialty training: (1) identity and professionalism (2), application and admission (3), support and social network (4), course content and expert guidance (5), assessments (6), microaggressions, and (7) perspectives on ideal GP-specialty training (Table 2).

**Table 2:** Key themes in participants’ responses

Themes	Descriptions
1. Identity and professionalism	Exploring the multifaceted nature of participants’ professional identities, based on the interviews’ identity card Sect.
2. Application and admission	Participants’ experiences and opinions related to the application and admission process
3. Support and social network	Support systems and social networks that participants relied on throughout their educational journey
4. Course content and expert guidance	Participants’ diversity, equity, and inclusion (DEI)-related perspectives on educational content and guidance
5. Assessments	Participants’ experiences and ideas regarding assessments, including examinations and evaluations, and their impact on their educational and professional trajectories
6. Microaggressions	Encountered instances of microaggressions, throughout the educational structure and their impact on participants’ well-being, educational outcomes, and professional opportunities
7. Perspectives on ideal GP-specialty training	Participants’ visions for ideal GP-specialty training, offering improved inclusiveness

### Identity dynamics and professionalism

From a set of nineteen options, participants selected identity cards representing their migration background, family history, ethnicity, and learning style, reflecting the aspects that shaped their professional journeys. They highlighted the challenge of being raised with norms and habits different from those of their Dutch-origin peers and their struggle when teachers and GP-supervisors misunderstood their behavior rooted in their specific backgrounds.

*‘Over time*,* I came to recognize that a significant part of my professional behavior was shaped by my upbringing*,* which differed markedly from that of my Dutch peers raised with Dutch norms. For instance*,* they naturally developed assertiveness to express their opinions’.* (participant 2, female, first-generation trainee)

*‘I might approach certain situations differently. I’m not referring to medical knowledge*,* where my performance is excellent*,* and patients express high satisfaction. I just meant*,* teachers might think that I reflect or think differently*,* or that I don’t make myself adequately visible…’* (participant 11, male, second-generation, dropped out).

*‘At times*,* maybe wrongly*,* I feel that teachers truly don’t comprehend me. Yes*,* that they lack a proper understanding… if they had just shown interest*,* I could have explained things to them more effectively.’* (participant 5, male, first-generation, alum).

Especially in situations requiring abstract norms, they emphasized the importance of concrete examples for better understanding.

*‘…for instance*,* when I have to delve into the patient’s question behind the question. What should I do in such a situation*,* what tools do I need? It was immensely helpful when the teacher suggested simply focusing on ICE: ideas*,* concerns*,* expectations… suddenly things clicked. That was the key: when dealing with abstract concepts*,* I require concrete examples to fully grasp and apply them.’* (participant 6, female, first-generation trainee).

Despite these challenges, participants viewed their multicultural backgrounds as an asset for understanding diverse patients rather than an obstacle to professionalism.

*‘…particularly when it comes to vulnerable individuals from diverse backgrounds who struggle to express themselves adequately and may not fully comprehend the doctor. I believe that due to my background*,* I have a more nuanced understanding for them than my Dutch colleagues. I find it truly rewarding to grasp people’s concerns and their cultural nuances.’* (participant 4, female, second-generation alum).

Moreover, they highlighted the lasting impact of discriminatory experiences in earlier education on their self-esteem.

*‘Previous situations of discrimination have made me wary*,* more on my guard. I thought it might not always be easy in this training program either.’* (participant 8, male second-generation trainee).

*‘I’m used to people having a standard impression of me as that outsider. Then*,* I find myself exerting extra effort*,* ten times over*,* to show them my true capacities.’* (participant 7, female, second-generation trainee).

Furthermore, they noted the effect of bilingualism on their Dutch reading skills, leading to slower reading and occasional misunderstanding of test questions due to a single unclear word.

*‘Typically*,* when being bilingual*,* you tend to grasp the essential meaning between the lines rather than literally translating… while unfamiliar words might not pose a significant hurdle in everyday conversations*,* they become problematic in test situations or when addressing patient needs. Learning the importance of seeking clarification when a patient uses unfamiliar language was a valuable lesson for me.’* (participant 14, male, second-generation trainee).

### Application and admission

Participants expressed feeling welcomed and respected during the admission process.

*‘I earnestly hoped for admission*,* concerned by past setbacks in my academic journey. The immediate acceptance was a relief. Experiencing success just by being myself was a pleasant and unexpected surprise.’* (participant 3: female, second-generation migrant trainee).

However, they emphasized disparities in admission interviews, especially concerning questions they deemed uncommon for their majority peers. This perception raised concerns about the committee’s unfamiliarity with their backgrounds or potential bias, leading to inappropriate and unjust focus within the application process and undermining recognition of their qualifications.

*‘Some committee members asked*,* “How will you integrate your religion into your work during consultation hours?” … I doubt my colleagues of Dutch origin would have faced similar inquiries. No. No. No.’* (participant 7: female, second-generation migrant trainee).

*‘… also*,* there were GP-supervisors who openly admitted: in all honesty*,* we have never had a trainee with an Islamic migration background or someone wearing a headscarf.’* (participant 4: female, second-generation migrant trainee).

### Support and social network in the educational environment

Participants noted positive interactions with teachers and peers, fostering a supportive learning environment that contributed to their professional growth and enhanced feelings of safety and inclusion. They valued these relationships for their genuine interest and support.

*‘So far*,* I have experienced a safe learning environment in the GP-training clinic and GP-specialty institute. The in-faculty teachers and in-clinic supervisor created a secure atmosphere where I could discuss everything. Sure*,* the trainee’s efforts are essential for professional growth*,* but we need a secure space to undertake these efforts … sometimes*,* it’s crucial to have someone who sees and appreciates you; a role model in that moment. Yes.’* (participant 7: female, second-generation migrant trainee).

*‘I didn’t feel that they* (the teachers) *see me as “that one foreigner”. No*,* not at all*,* we have a genuine connection and good contact.’* (participant 1: male, first-generation trainee).

Despite valuing individual relationships, participants felt unheard and often disconnected from in-faculty and in-clinic feedback due to organizational miscommunication. Critical decisions, like education termination or reintegration after illness, lacked discussion opportunities.

*‘… I did not recognize his feedback. I sought to discuss this with my teachers*,* but they blindly followed my GP-supervisor’s view. I just didn’t feel heard…’* (participant 4: female, second-generation migrant alum).

*‘… I had to go to the training institute on Friday*,* where they announced that my training was being terminated. Yes*,* without any genuine opportunity to discuss this.’* (participant 11: male, second-generation, dropped out).

*‘In my reintegration after illness*,* I repeatedly expressed concerns about the challenges I faced. I felt this was going wrong*,* but my pleas seemed unheard … and I had to shout out that I needed support in rebuilding my workload gradually.’* (participant 10: female, first-generation alum).

In this light, participants observed inadequate inquiry by management and staff into educational barriers for migrant trainees, leaving their concerns unaddressed. The organization’s inexpedient attention to specific barriers allowed exclusion or microaggressions to go unnoticed in structural policies and risk analyses.

*‘…no official representing the organization has ever asked me*,* as a migrant trainee*,* if I had experienced any specific barriers in which I needed institutional support.’* (participant 5: male, first-generation migrant alum).

*In the introductory session*,* they had us participate in a round where we stood in a place*,* representing the distance of our birthplaces from the institution. Yes… being the sole participant born outside the Netherlands*,* I painfully felt out of place. While I acknowledge no harm was intended*,* they need to step into my shoes to genuinely understand how such thoughtless activities can be excluding and offensive.’* (participant 3, female, second-generation trainee).

Despite sometimes feeling excluded, participants remained committed to pursuing their chosen profession.

*‘I will never be part of that Dutch white world*,* but I am determined to become part of the GP world.*’ (participant 12: female, second-generation trainee).

### Course content an expert guidance

Concerning course content and delivery, participants appreciated small-group in-faculty and one-to-one in-clinic learning. However, they raised concerns about the curriculum’s lack of diversity and the predominant ‘white’ representation among teachers, GP-supervisors, and peers.

*‘In the matching carousel you meet all in-clinic supervisors and in-faculty teachers and also the supervisor with whom you will ultimately be placed in the first year of training. Yes*,* of course they are all white and Dutch with mainly white roots’.* (participant 6: female, first-generation trainee)

They highlighted a risk of insufficient reflexivity due to monoculturalism, noting inadequate reflection among teachers and GP-supervisors on guiding trainees from diverse backgrounds and those who struggled in managing care for patients from different cultures.

*My GP-supervisor should have done a little more self-reflection on our gap of understanding … but I’ll leave that open*,* I reflected on it and learned from I and it’s about my development; if he chooses not to reflect*,* that’s up to him.* (participant 2: female, first-generation trainee)

*‘Lots of questions about these patients with habits unfamiliar to them*,* lots… “What is that like*,* and how should I handle this?” Yes*,* that really… yes*,* it just shocked me.’* (participant 5: male, first-generation alum).

A recurring theme was participants’ sense of alienation from peers, feeling ‘the deviant other’ when singled out as experts on habits or values within their ethnic group. Although unsettling, some reluctantly accepted this role and offered expertise as a compromise for the greater good.

*‘Of course it feels meaningful to contribute from my perspective*,* but if the subject is so significant*,* it should not be my job to step out of my comfort zone as a trainee. To me*,* it is doubly challenging*,* yes.’* (participant 14: male, second-generation trainee).

*‘If they did not ask*,* many more prejudices and incorrect stereotypes would persist.’* (participant 9: female, first-generation, alum).

Participants valued course content on intercultural competencies but were disappointed by its superficial and stereotypical nature. They also felt some teachers and GP-supervisors lacked sufficient understanding.

*‘…a teacher*,* a GP well-versed in migrant care*,* joins*,* and one hopes for valuable insights … but then he presents a stereotypical case of an Islamic patient refraining from shaking hands deemed respectful in Islamic norms but not in Dutch customs. Yet*,* this teacher neglects the shared value of being respectful by hastily passing judgment rather than seeking an understanding of backgrounds’.* (participant 3: female, second-generation trainee)

Participants grappled with navigating unspoken norms in formal and informal educational contexts, citing examples that mirrored prevailing cultural values and attitudes. This struggle adversely affected their in-clinic and in-faculty learning experiences, risking unjust perceptions of underperformance due to deficient intercultural understanding among teachers and supervisors.

*‘In my culture*,* I learned to carefully oversee the situation around me before reacting. My supervisor was fiercer; she did not mince her words. She directed me to express my opinion her way*,* but I was educated the opposite. She found my attitude too expectantly and inappropriate.’* (participant 11: male, second-generation, alum).

*‘My GP-supervisor found it problematic; he had never trained anyone wearing a hijab before… he was a bit like*,* “How will my patients react*,* as they are not used to this?”.’* (participant 4: female, second-generation, alum).

*‘Sometimes*,* I did not comprehend people’s reactions and felt desperate*,* wondering what I did wrong. When I sought guidance from my teacher*,* she couldn’t grasp my problem. Instead*,* she suggested asking another hijab-wearing girl with a migration background in a different GP-trainee group. So she said*,* maybe ask her how she deals with that. In the end*,* it didn’t help me much*,* like hitting a wall’.* (participant 13, female, second-generation alum)

*‘It was not something I had recognized as important*,* and I was unaware that the training expected this of me. Later*,* I discovered that this is considered important - these are unwritten rules - a Dutch-raised trainee would be much more likely to have learned these norms from home.’* (about actively engaging in an educational conversation; participant 2: female, first-generation trainee).

### Assessments

In in-clinic and in-faculty assessments, participants navigated the delicate balance of conforming to mainstream norms while being instructed to express their authentic selves. This challenge was compounded by norm ambiguity and the complex interplay of dependency on educator assessments while adhering to the requirement of shaping their unique professional identities.

*‘The teachers urged me to express my true self. So*,* I made a continuous effort to be my authentic self. However*,* what I presented never seemed to meet their standards.’* (participant 12, female, second-generation trainee).

Discussing assessment tools, participants noted that the Competency Assessment List, while seemingly straightforward, revealed subjective elements like assessors’ preferences and goodwill factors. They found the National GP-Knowledge Test norms more explicit and transparent.

*‘A competency measuring instrument appears logical*,* but it constrains you within its framework. It aims for uniformity*,* but overlooks the individual differences among trainees. Personally*,* I don’t find the Competency Assessment List useful; it is too rigid*,* lacking the necessary nuance and causing false sense of representation.’* (participant 11: male, second-generation, dropped out).

*‘I think the National GP-Knowledge Test is a nice instrument to measure how your knowledge is increasing. So*,* as a progress test*,* this is an excellent method.’* (participant 2: female, first-generation trainee).

*‘Certainly*,* the National GP-Knowledge Test employs a testing format familiar from my educational experiences. So for me*,* it is very concrete. I can just navigate fine with it.’* (participant 6: female, first-generation trainee).

Participants noted that in-clinic assessors operating independently introduced bias risks due to potential personal preferences, unlike the collaborative approach of in-faculty assessments. This divergence raised concerns about fairness and objectivity.

*‘My next GP-supervisor received the transfer report from the supervisor who deemed my performance unsatisfactory. There were no specific feedback points provided… maybe personal issues had influenced the assessment.’* (participant 10, female, first-generation trainee).

### Microaggressions

Throughout their educational journeys, participants encountered microaggressions as significant obstacles to their professional development. They did not always immediately recognize these subtle put-downs as external factors. Initially, this led to feelings of unease and uncertainty, hindering them from fully expressing themselves, which is crucial for successful progress in GP-specialty training. Through repeated incidents and discernible patterns, they came to identify these behaviors as microaggressions originating from others, profoundly impacting them.

Participants emphasized teachers’ negative stereotyping in case presentations and the normalization of racism in the educational setting, exacerbated by their failure to condemn discriminatory incidents.

*‘During one course*,* a teacher presented a case about a boy with a specific skin condition. In describing his background*,* she referred to him as ‘such Moroccan brat*,*’ an entirely irrelevant characterization to the case.’* (participant 8: male, second-generation trainee).

*‘There you are*,* in her class*,* with thirty other trainees and a few more teachers*,* a heavy silence hangs in the air. No one speaks up. Not a single teacher or trainee utters a word*,* as if it’s the most ordinary situation. It’s disheartening*,* making you wonder*,* “What am I hearing? What is she saying?” This scenario happened repeatedly*,* amplifying my internal struggle.’* (participant 6: female, first-generation trainee).

They felt excluded by in-clinic supervisors expressing surprise at minority trainees speaking flawless Dutch.

*‘I have had many unpleasant discriminatory experiences*,* where do I start… in my second year of training*,* while introducing myself to the assistants at my new internship*,* the GP-supervisor came over and I introduced myself to him as well. With widened eyes*,* he remarked to others*,* “He speaks Dutch.” Inside I completely broke down. ! Disbelief! Especially considering my academic background and pursuit of specialization…’* (participant 8: male, second-generation trainee).

*‘… and they say*,* ‘Wow*,* you speak Dutch so well’… Then I think*,* yes*,* it’s a compliment that I speak Dutch fluently*,* but there is always… a prejudice underneath? That’s what I wonder*,* and I sense that*,* how people deliver those compliments… It doesn’t feel like a compliment actually. Like*,* I don’t really belong here.’* (participant 2: female, first-generation trainee).

*‘… and that is what I feel when people give me those compliments… I convince myself just to see it as a compliment*,* but I know there is a… eh*,* a hidden message of not belonging… It actually doesn’t feel like a compliment*,* to be honest’* (participant 1, first-generation trainee).

Participants highlighted instances of teachers openly expressing prejudices against women and foreigners, promoting the adoption of Dutch habits without meaningful dialogue.

*‘… then one GP-supervisor stated: ‘if someone refuses to shake my hand; I certainly have an opinion about that*,* that’s just how we do this in the Netherlands and they have to adapt.” The intensity of this statement left me wondering: “wow*,* where does this sentiment come from?”. As I felt a rising discomfort within me*,* I couldn’t help but thinking “damn it”.’* (participant 3, female, second-generation trainee).

*‘In the matching process*,* one GP-supervisor openly admitted that wearing a hijab would not align with their organizational culture. He expressed concerns that my headscarf would create barriers with his patients accepting me as a professional.’* (participant 7, female, second generation trainee).

Participants often faced subtle incidents, like ‘us-and -they’-language, generalized attributions to their ethnicity, subtly racially insulting jokes, biased assessments against minority trainees’ clinical mistakes, questioning of their judgment based solely on cultural backgrounds, inappropriate inquiries about upbringing or family history, and understated remarks regarding appearance, hobbies, or beliefs. Despite teachers and assessors maintaining verbal correctness, participants frequently experienced an ‘othering’ effect due to non-verbal rejection.

*‘… we recently had a course about migrants … I am the only one with a different background. That’s always a laugh; not really though. Then I feel addressed again*,* thinking*,* “Oh my… I don’t want everyone to stare at me again to find answers to their dilemmas”. I find that difficult.’* (participant 3: female, second-generation migrant trainee).

*‘… during the lessons*,* those about diversity*,* when my opinion is often asked*,* I feel comfortable… wondering why only I should be an expert in this subject*,* why don’t they educate themselves?’* (participant 7: female, second generation trainee).

*‘Unfortunately*,* it’s an undeniable reality*,* my skin color catches their eyes. The heightened attention is palpable*,* it leaves me consciously pondering*,* “How am I perceived? Have I said enough*,* asked the right questions? What judgments linger?”* (participant 11: male, second-generation trainee).

*‘I always try to shield myself by consistently putting a positive spin on things. Uhm… when someone unrelated asks about my origin*,* I mention my place of residence: [Dutch city]*,* attempting to downplay a sense of exclusion…’* (participant 2: female, first-generation trainee).

*‘Verbally*,* things may seem fine*,* but non-verbally*,* I sense… It might sound harsh*,* but it feels like they’d rather let me go than include me. Yes*,* that’s how it feels.’* (participant 7: female, second-generation trainee).

These experiences left a lasting sense of not belonging and discomfort, deterring them from seeking attention as emerging professionals and prompting deliberate efforts to maintain a low profile.

*‘I feel that I must be careful not to stick my head too far above ground level. Before you’ll notice*,* you’ll be under their microscope.’* (participant 8, male, second-generation trainee).

Balancing these emotions with the demands of GP-specialty training deeply affected participants’ sense of safety. While some found comfort in supportive individuals who created safe spaces, many reported fatigue, diminished self-esteem, burnout, illness, and depression. Consequently, some underperformed and, in some cases, failed to complete their postgraduate training.

## Perspectives on ideal GP-specialty training

### Application and admission / Support and social network

Participants envisioned an ideal GP-specialty training that values each trainee’s uniqueness and avoids seeing diversity as an obstacle. They emphasized inclusive participation, urging proactive efforts to engage minority trainees in policy-making committees and making every voice heard.

*‘… I believe there’s room for embracing individuality*,* incorporating personal character and cultural values*,* so that we can learn from each other more effectively.’* (participant 3: female, second-generation trainee).

*‘Attentively listen to minority trainees*,* delve into their backgrounds*,* and make an effort to understand them for the enhancement of the training program’s quality.’* (participant 11: male, second-generation, dropped out).

### Course content and expert guidance / Microaggressions

The ideal training would feature a robust DEI-policy with decisive action against (un)conscious biases and discriminatory incidents. Participants urged mandatory staff training to enhance DEI-awareness, emphasizing the avoidance of stigmatizing case studies and generalized learning style promotion.

*‘… a zero-tolerance policy for racism in the training program*,* even if it is implicit. So*,* scrutinize policies that may indirectly perpetuate racism*,* leading to*,* for instance*,* fewer minority applicants entering training… I mean also develop an eye for subtle forms of discrimination.’* (participant 8: male, second-generation trainee)

*‘Only few teachers are skilled in diversity*,* for most of them*,* it isn’t their focus. That should be improved*,* there is a need for comprehensive training for all teachers and GP-supervisors.’* (participant 5: male, first-generation alum).

*‘In Eastern cultures*,* where I come from*,* we approach feedback differently. Teachers may perceive a challenge in my handling of feedback*,* and I realize that I need to learn the Dutch way. But it’s also crucial for them to recognize that my learning might manifest differently than what they are accustomed to.’* (participant 5: male, first-generation alum).

### Assessments

Participants emphasized the importance of clear standards and objectives in assessments. They endorsed concise language in test questions, eliminating double negatives in the National GP-Knowledge Test, and promoting open-ended questions over multiple-choice formats. Lastly, the ideal system would prioritize individual mentorship for trainees who desire such support.

*‘GP-supervisors or in-faculty teachers as role models or mentors who simply share their experiences. I think it is important that during the training there is someone*,* perhaps with a migration background or someone interested in diversity*,* who they can fall back on.’* (participant 4: female, second generation alum).

*Teachers and GP-supervisors*,* they are all so white. So*,* how can they make a connection with the minority trainees? GP-specialty training institutes can also take their own initiative*,* right? Why is it that minorities don’t speak up or take more active roles? Our hesitancy may be rooted in past struggles*,* but letting go of the effort is regrettable.’* (participant 10: female, first-generation alum).

## Discussion

### Summary of findings

Fourteen interviewed GP trainees and recent alums from ethnic minority backgrounds encountered educational barriers due to ‘white’ predominant teacher misunderstandings and organizational inattention to minority trainees. They navigated between mainstream norms and nurturing authentic professional growth. Various educational aspects presented distinct challenges and opportunities, with settings farther out of sight from institutional staff being more prone to microaggressions. Despite positive individual relationships, participants experienced minimal organizational support in conflicts with in-clinic supervisors, stemming from in-faculty teachers’ loyalty to these supervisors. Assessments, particularly in professional behavior and communication, heavily relied on individual supervisors, leaving them vulnerable to loosely defined criteria. Conversely, strict assessment standards limited the expression of individual styles, hindering personal and professional development. Participants noted hidden subjective elements in apparently neutral assessments, posing bias risks. Throughout their educational journey, participants faced various forms of microaggression. They envisioned ideal GP-specialty training focused on each trainee’s uniqueness, inclusive participation, robust DEI-policy, and individual mentorship while advocating for clear standards, concise test question language, and bias elimination in assessments.

### Comparison to existing literature

This study explores minority GP-specialty trainees and recent GP-graduates educational journey, complementing previous research on minority undergraduate medical students and clinical trainees [18, 20, 36, 49–51]. The findings align with existing evidence on the significance of inclusive learning environments for well-being and academic achievement [15, 38].

Comparison to existing literature *per educational aspect* outlined in our results:

### Application and admission

Consistent with US findings linking admission stress to prior discrimination experiences [29], our participants expressed concerns about racial stereotyping and the majority´s resistance to social change [32]. They encountered biases from committees unfamiliar with their backgrounds, compromising fair qualification recognition. The moderate reliability of Dutch disciplined-based admission interviews [30], the UK’s competency-based selection [31], and Denmark’s Multiple Mini Interviews [63] accentuated these concerns.

### Support and social network in the educational environment

The participants’ perceptions of their educational environment revealed boundaries and support mechanisms that tend to go unnoticed within organizational processes [64]. Their feelings of alienation and being unheard, stemming from the curriculum´s limited diversity and predominant ‘white’ representation with a tendency of stereotyping and insufficient multicultural reflexivity, resonate with literature on the impact of othering in medical learning environments [33, 34, 39]. The complexity of these educational structures [65] and unspoken norms may contribute to the implicit exclusion of ethnic minority professionals and trainees throughout their careers [44]. Despite these challenges, participants valued positive relationships with genuinely engaged teachers and collaborative learning with peers. Studies underline the importance of mentors who show interest and belief and provide development opportunities as critical predictors of career success [33, 66].

### Course content an expert guidance

Consistent with PIF (Professional Identity Formation) research among minority medical trainees [35, 36], our participants perceived constraints in their professional identity development due to inadequate space for diverse norms and learning styles, compounded by discriminatory experiences and stereotyping course content.

### Assessments

Particularly in assessments, participants intricately balanced personal and professional identities, positively integrating or downplaying them for success. Literature confirms that this complexity, involving assessor dependency and subjective preferences, poses barriers to full professional expression for minority students [35], risking feelings of self-doubt and isolation [37].

### Microaggressions

Participants struggled to recognize microaggressive behaviors as a cause for their unease. Accordingly, they emphasized that educational organizations may face challenges detecting these instances, potentially hindered by unintentional bias. Sue’s Taxonomy of Microaggressions offers a valuable framework for recognizing and addressing these behaviors(Fig. 1) [62]. By categorizing microaggressions into microinvalidations, microassaults, and microinsults, this taxonomy provides a structured approach for understanding how these behaviors contribute to exclusion and hinder trainees from fully expressing themselves, crucial for fostering inclusivity in postgraduate medical education. Our participants encountered all three forms of microaggressions throughout their educational journey, with microassaults being slightly less common.

Consistent with the literature, microaggression experiences exacerbated feelings of othering and integration challenges [15, 23, 40] and negatively affected our participants’ professional performances [18, 38, 39]. Throughout their GP-education, participants frequently felt excluded, lacked a sense of belonging, and felt perceived as deviant. Whether in-faculty or clinical settings, they spent a significant amount of time contending with microaggressions, leading to persistent feelings of loneliness and disconnection [41–43]. Although less frequent, microassaults towards ethnic minorities in medical education occur [62], with our study’s participants noting instances of verbal aggression and intimidation.

### Perspectives on ideal GP-specialty training

Aligning with the literature, participants considered confidence in novel approaches [36] and the target group’s needs and aspirations [67] essential for creating an inclusive GP-specialty training. They advocated for diverse committees, organization-wide bias training, blinded applications, and eliminating biased metrics, which resonates recent US findings on inclusive measures in clinical orthopedic education [68]. Participants envisioned an ideal training that values each trainee’s uniqueness and diversity as assets. They called for a clear DEI-policy condemning racism and stereotyping, DEI-training for all staff, clear assessment standards, open-ended test questions in unequivocal language, and targeted mentorship as crucial elements for an inclusive learning environment.

### Strengths and limitations

Our open-ended, trainee-centered approach [69] facilitated rich narratives with numerous examples of lived experiences, offering comprehensive insights into diverse trainee perspectives throughout their educational journey in GP-specialty training. The deductive methodology aligned outcomes with two established frameworks for better understanding.

Despite the modest sample size, saturation was achieved, covering all educational stages, including recent graduates. However, limitations may exist, as participants driven by their specific interests may not fully represent the broader minority GP-trainee population. Transferability limitations may arise from contextual variations, such as differences in educational emphasis and cultural factors. However, accurately describing our study’s setting and leveraging similarities across postgraduate medical education settings helps maintain applicability to settings sharing these characteristics. Classifying findings by educational aspects provides insights for comparable programs and aids in identifying overarching concepts. Additionally, linking participant perspectives to relevant literature and frameworks enhances understanding of underlying dynamics.

### Implications for future research and practice

Our findings underscore the prevalence and diverse forms of microaggressions and challenges faced by ethnic minority trainees in various GP-specialty training aspects (such as course content, expert guidance, support, social network, and assessment and admission bias), emphasizing the need for increased awareness and cultural sensitivity in training environments.

For a more nuanced understanding of the complex educational context in GP-specialty training, future research could employ mixed methods involving quantitative designs to assess the significance of various factors reported by participants. Additionally, qualitative exploration of minority trainees’ coping strategies would be valuable. Implementing a participatory action approach with sounding board groups can help develop and continually adjust broadly supported inclusive strategies within the organization.

## Conclusion

Despite valued personal connections within their educational environment, ethnic minority GP-trainees in the Netherlands face significant educational challenges, including constraints in professional identity development, assessment and admission bias, lacking support networks and guidance, stereotyping and subjective elements in course content and assessments, and microaggressions, leading to burnout, depression, and underperformance. While participants consider their multicultural backgrounds as assets, challenges arise from diverse upbringings and discriminatory experiences. Although generally welcoming, admission processes and organizational support raise concerns about bias due to unfamiliarity with diverse backgrounds and a lack of diversity.

For ideal GP-specialty training, participants recommend focusing on each trainee’s uniqueness, proactive engagement in inclusive strategies, robust DEI-policies with decisive anti-discrimination actions, individual mentorship, transparent educational standards, concise language usage, and open-ended test questions. They emphasize the necessity of mandatory staff DEI-training programs to eliminate assessment and guidance bias.

Further research is recommended to explore the significance of reported factors, minority trainees’ coping strategies, and the development of inclusive strategies within organizations.

## Supplementary Information

Supplementary Material 1.

## Data Availability

The datasets generated and analyzed during the current study are not publicly available for individual privacy reasons but are available from the corresponding author upon reasonable request.
