# Supplementary material for: Exploring the educational journey: perspectives of ethnic minority GP-trainees in Dutch GP-specialty training - a qualitative interview study
Source: Int J Equity Health. 2024 Nov 28;23:253. doi: 10.1186/s12939-024-02341-x (PMC11603862; doi:10.1186/s12939-024-02341-x)
Supplement: Supplementary file 1 — Supplementary Material 1. [file 12939_2024_2341_MOESM1_ESM.docx]

**Appendix**

Topic list:

1. Personal background and its effect on experiences in the GP-specialty training
2. Progress in GP-specialty training and current status
3. Noteworthy experiences during GP-specialty training (both formal and informal interactions, influential individuals (role models), encountered barriers, and facilitators)
4. Perceived space for a unique professional identity forming
5. Specific experiences related to being part of a minority group (encounters with microaggressions, sense of belonging, and sources of support)
6. Interactions with assessors, insights on assessments, and thoughts on assessment tools
7. Ideas and suggestions for an ideal and more inclusive GP-specialty training program.
